# IoT-Based Heartbeat Rate-Monitoring Device Powered by Harvested Kinetic Energy

**DOI:** 10.3390/s24134249

**Published:** 2024-06-29

**Authors:** Olivier Djakou Nekui, Wei Wang, Cheng Liu, Zhixia Wang, Bei Ding

**Affiliations:** Tianjin Key Laboratory of Nonlinear Dynamics and Control, School of Mechanical Engineering, Tianjin University, Tianjin 300350, China; olivierdjakou@tju.edu.cn (O.D.N.); liucheng94@tju.edu.cn (C.L.); zhixiawang@tju.edu.cn (Z.W.); dingbei_784@tju.edu.cn (B.D.)

**Keywords:** biomedical signal processing, electromagnetic energy harvester, IoT server, kinetic energy harvesting, long-term ECG monitoring, quadratic non-linearity, Schenkel doubler, wearable IoT devices

## Abstract

Remote patient-monitoring systems are helpful since they can provide timely and effective healthcare facilities. Such online telemedicine is usually achieved with the help of sophisticated and advanced wearable sensor technologies. The modern type of wearable connected devices enable the monitoring of vital sign parameters such as: heart rate variability (HRV) also known as electrocardiogram (ECG), blood pressure (BLP), Respiratory rate and body temperature, blood pressure (BLP), respiratory rate, and body temperature. The ubiquitous problem of wearable devices is their power demand for signal transmission; such devices require frequent battery charging, which causes serious limitations to the continuous monitoring of vital data. To overcome this, the current study provides a primary report on collecting kinetic energy from daily human activities for monitoring vital human signs. The harvested energy is used to sustain the battery autonomy of wearable devices, which allows for a longer monitoring time of vital data. This study proposes a novel type of stress- or exercise-monitoring ECG device based on a microcontroller (PIC18F4550) and a Wi-Fi device (ESP8266), which is cost-effective and enables real-time monitoring of heart rate in the cloud during normal daily activities. In order to achieve both portability and maximum power, the harvester has a small structure and low friction. Neodymium magnets were chosen for their high magnetic strength, versatility, and compact size. Due to the non-linear magnetic force interaction of the magnets, the non-linear part of the dynamic equation has an inverse quadratic form. Electromechanical damping is considered in this study, and the quadratic non-linearity is approximated using MacLaurin expansion, which enables us to find the law of motion for general case studies using classical methods for dynamic equations and the suitable parameters for the harvester. The oscillations are enabled by applying an initial force, and there is a loss of energy due to the electromechanical damping. A typical numerical application is computed with Matlab 2015 software, and an ODE45 solver is used to verify the accuracy of the method.

## 1. Introduction

Nowadays in the context of COVID-19, wearable IoT devices play a crucial role in telemedicine since they can reduce risks of infections by remote assessment of vital human signs. The current commercial model of wearable IoT devices are powered by battery technology, but it has been recognized that there is an undeniable need for the energy requirement of ambulatory devices to be addressed [1,2,3,4,5,6]. Furthermore, wearable devices are embedded systems having no access to a charging source for their battery, which causes discontinuities to the monitoring process. In this regard, continuous monitoring of health data remains a considerable challenge for wearable IoT devices. This is despite the fact that battery technology has gone through considerable research and improvement [7]. In fact, the concern is to achieve both portability (small size) and operating time (back-up time). Analog integrated circuits and low-power microcontrollers (MCUs) are available for lowering the power needed by wearable devices. But achieving a low power demand by leveraging most of the latest design technologies without enhancing power management would not be efficient. There have been undertakings for developing IoT devices with low power demand in an attempt to improve the monitoring time, such as ECG data compression methods developed in [3,4,5], which use Fourier and wavelet transforms for compressing ECG signals and reduce the power required for transmission. An online dictionary-based approach presented in [3] further extends the compression algorithms to other bio signals such as respiratory rates (RESP) and photo-plethysmography (PPG) and quantitatively assesses the compression, reconstruction, and energy consumption performance of different schemes. Even though signal compression approaches can considerably lower energy demand for transmission, bio-signal quality and precision after compression still remains an open question. However, harvesting devices generating electric energy from the environment through direct energy conversion for biomedical devices have proven to be effective alternatives [8,9,10] which do not alter health data. Intensive studies of power harvesters using human body motion to power biomedical sensor nodes were carried out by Paulo and Gaspar in 2010 [11] and Jaeseok et al. in 2011 [12]. Much research has focused on remote heartbeat rate monitoring and some commercial devices powered by battery technology are available. A Hicardi system [13] is a mobile cardiac-monitoring device for the instantaneous monitoring of cardiac, respiratory, and temperature activities, and it sends data to a secured cloud. It is a battery-powered device, and Wi-Fi transmission is used for real-time communication with a remote server. The Philips Page Writer TC10 cardiograph [14] is a small, embedded ECG system including various features such as: compact size and light-weight design, bidirectional network communication using secure, wireless connectivity based on LAN protocol, an LCD screen for visual guidance and monitoring of ECG graphs, and a printer for providing hard copies of heartbeat rate record. A Holter monitor [15] is a battery-operated, wearable monitor for continuous recording of heart activities over 24–48 h. The Holter monitor uses Wi-Fi transmission for sending heart data to a cloud server. Other innovative technologies for remote health data monitoring include the following: remote monitoring of blood oxygen saturation using a contactless mHealth solution [16], which processes short facial video recorded with any common mobile device for estimating oxygen saturation in the blood (SpO2) based on remote photo-plethysmographic signals (rPPGs). A wearable electrocardiogram telemonitoring system for atrial fibrillation detection [17], which uses a designed, wearable ECG patch for collecting ECG signals and forward them to smartphones via Bluetooth transmission. An android application was developed for displaying the ECG waveforms on smartphones and forwarding health data to the cloud server every 30 s. A smart, low-cost, wearable device has been developed by Eesha Tur Razia Babar et al. for remote patient monitoring; the device continuously measures vital sign parameters such as: temperature, pulse rate, and pulse oxygen concentration using microelectronic devices and forwarding them to remote medical staff through Wi-Fi transmission [18].

This article presents an IoT-based heartbeat rate-monitoring device which is powered by battery. In comparison with the existing types of ECG monitoring devices, the practical application of harvesting kinetic energy from daily human activities to sustain the power of biomedical devices is demonstrated in this study. It enables us to create a more energy-efficient device, addressing the gap by developing a green device that can replace the current models. The ECG device is a stress or exercise type, which can continuously monitor the heartbeat rate of patients during normal daily activities such as walking, running, and jumping. The experimental prototype monitors heartbeat rate with electrocardiogram electrodes (AD8232), the data are processed with the microcontroller (PIC18F4550) and sent to the IoT server through the Wi-Fi serial transceiver device (ESP8266). This research makes use of a Matlab server which is accessible by creating a personal space for storage on cloud (https://thingspeak.com, accessed on 25 June 2024). The heartbeat rate can be accessed by any mobile device, cardiology hospital, or any other off-site scientist. Moreover, it is essential to improve the monitoring time of the device and enhance the device with the harvester supplying perpetual energy. This function is assumed by an electromagnetic energy harvester which collects vibration energy from human motion and transforms it into electricity for powering the device. Due to the non-linear interaction force of the magnets, the dynamic equation illustrating the motion of electromagnetic harvester has an inverse quadratic non-linearity, which relates the magnetic force to the separation distance between the magnets. The same approach was described by other authors [19,20].

This study considers the case for electromechanical damping, the quadratic non-linear force of the magnet is approximated in this research using MacLaurin expansion, which enables us to provide an exact solution to the dynamic equation using classical methods for dynamic equations [21] and find the suitable parameters for the harvester. A typical numerical application is done in Matlab 2015 and ODE 45 solver to verify the effectiveness of this method. The bridge rectifier and voltage multiplier are made of low power switches (1N4001) and capacitors.

## 2. Architecture of IoT-Based ECG Monitoring Device Powered by Energy Harvester

**Figure 1:**
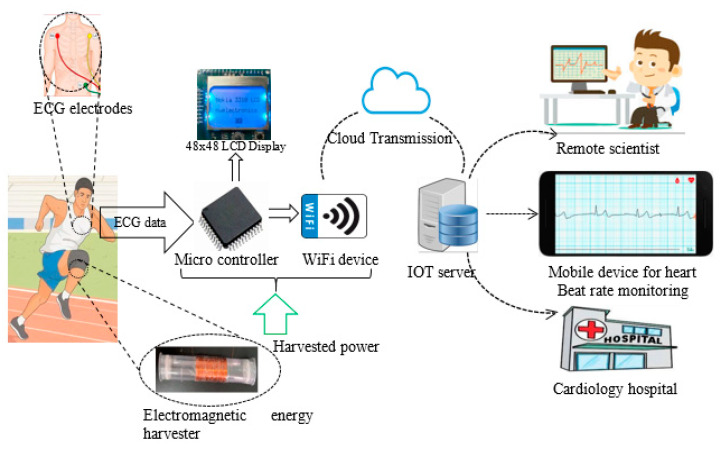
Architecture of IoT based ECG monitoring device powered by energy harvester.

In this section, the architecture of the IoT heartbeat rate-monitoring device with the energy harvesting system, which enables long-term monitoring of out-patients living with unstable health conditions and necessitating continuous monitoring, is presented,. As illustrated in (Figure 1), the device consists of five typical parts including the IoT server, the microcontroller, Wi-Fi serial transceiver for forwarding the health data to the cloud server, the heartbeat rate sensor, and the electromagnetic energy harvester. Further details about the IoT server, microcontroller, Wi-Fi serial transceiver, and heartbeat rate sensor are provided in the experimental section.

▪ Electromagnetic energy harvester (EMEH)

**Figure 2:**
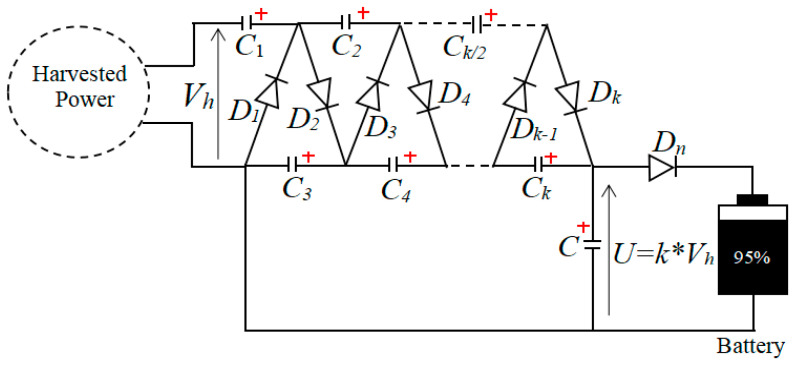
Voltage multiplier and rectifier.

The EMEH is made of a coil-magnet. It collects vibration energy from daily human activities such as walking, running, and jumping to generate electricity. Neodymium magnets are chosen for their high magnetic strength, versatility, and compact size, which make them suitable solution for applications requiring a strong magnetic field [22]. The EMEH consists of two end magnets and a middle magnet with each pole facing the opposite pole of each end magnet. The coil is wrapped around a cylindrical tube inside, to which the magnets are aligned. The harvester is attached to the lower extremity of the body or arms. Human motion induces the displacement of the central magnet of the harvester, which causes changes in the magnetic flux around the coil and generates electricity based on the principle of electromagnetic induction. The voltage multiplier and rectifier in (Figure 2) is made of ultra-low power electronic switches and electrolytic capacitors. It is designed to get a multiplier ratio k = 16. It is used for rectifying the power from the harvester and adapting the harvested voltage to the battery voltage for charging. Both first switches $D_{1}$ and $D_{2},$ with capacitor $C_{1}$, form the first Schenkel doubler [23], which charges the capacitor $C_{3}$ at a maximum value of 2 × $V_{h\;max}.$ $D_{3}$ and $D_{4}$, with capacitor $C_{2},$ form the second doubler, charging the capacitor $C_{4}$ at a maximum value of 4 × $V_{h\;max}$. $D_{k-1}$ and $D_{k},$ with capacitor $C_{k/2},$ form the last doubler, charging the capacitor $C_{k}$ at a maximum value of $k\times V_{h\;max}$. The capacitor C is used for filtering the multiplied voltage, and the voltage across the capacitor ${U=k\times V}_{h}$ is used for charging the battery. The switch $D_{n}$ limits reverse current from battery. The harvested power is used for charging the battery of the wearable device and improves the monitoring time.

Electronic design of the whole experimental setup including microcontroller, heartbeat rate sensor, Nokia LCD, Wi-Fi serial transceiver, and power conditioning (for harvested energy) is first made in ISIS 8.0.1 professional software (Appendix A). “C” program is used for interfacing with ISIS 8.0.1 professional software and performing virtual simulation before manufacturing.

▪ Printed circuit board design

**Figure 3:**
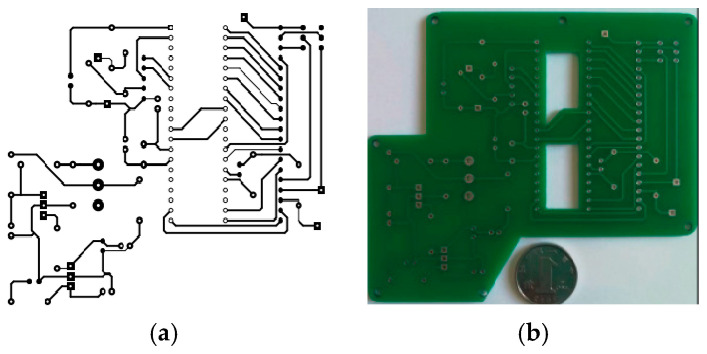
Printed circuit board for IoT-based heart rate-monitoring device: (**a**) designed board, (**b**) manufactured board.

ARES from Proteus 8.0.1 software is used for designing the printed circuit board (PCB). A Pickit 3 device is used for transferring the program to the experimental prototype. (Figure 3a) below shows the designed PCB board and the printed board (Figure 3b).

## 3. Dynamic Analysis of Electromagnetic Energy Harvester

**Figure 4:**
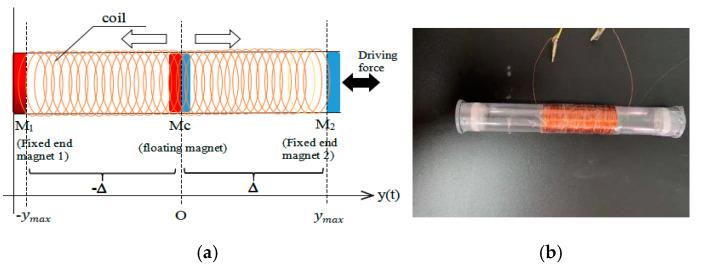
Design (**a**) and physical model (**b**) of electromagnetic harvester.

In this section, a special oscillating system is studied, which consists of two fixed end magnets and a central floating magnet as illustrated in (Figure 4). The central magnet is oriented for repulsion, provoking A non-linear magnetic field inside which appear different types of oscillations of the central magnet. The magnet body is subjected to the non-linear magnetic field of the permanent magnets which have an inverse quadratic dependence on distance.

The following assumptions hold with this study:

- The magnetic field produced by the permanent magnets is non-conservative and there is a loss of energy in the magnetic interaction due to electromechanical damping;
- The oscillations start by applying an initial force on the system;
- The applied force on the cylindrical tube by human limbs follows simple harmonic motion on the form Fcos⁡ωt.

Based on these assumptions, the law of motion for the general case study is found.

The ideal expression of magnetic force is considered in this study. The well-known classical expression of magnetic force is shown below [24,25]:

(1) $$F_{m}=\frac{\sigma_{m}}{{\left[y_{\left(t\right)}-\Delta \right]}^{2}},$$

where $F_{m}$ is the magnetic force, $\sigma_{m}$ is the magnetic constant related to the strength of the magnet, *y* is the distance between magnets, and ∆ is the change in the distance between magnets.

The relationship between the force acting on the central magnet and the displacement is shown in the below Table 1.

The implied dynamic equation of the harvester is shown below:

(2) $$m\frac{d^{2}y(t)}{dt^{2}}=-c\frac{dy(t)}{dt}+\frac{\sigma_{m1}}{{\left[\Delta +y(t)\right]}^{2}}-\frac{\sigma_{m2}}{{\left[\Delta -y(t)\right]}^{2}}+F\cos\omega t,$$

where *c* is the electromechanical damping constant, *m* is the mass of the floating magnet, and $\sigma_{m1}$ and $\sigma_{m2}$ are constants related to the strength of magnet 1 and 2, respectively.

The magnetic force in (2) is linearized by MacLaurin expansion to the first order and it is obtained through the following equation:

(3) $$\frac{\sigma_{m1}}{{\left[y(t)-\Delta \right]}^{2}}-\frac{\sigma_{m2}}{{\left[y(t)+\Delta \right]}^{2}}\approx \frac{\sigma_{m1}-\sigma_{m2}}{\Delta^{2}}-\frac{2(\sigma_{m1}+\sigma_{m2})}{\Delta^{3}}y(t)$$

Substituting (3) into (2), the corresponding dynamic equation for the harvester is obtained in Equation (4) below:

(4) $$\frac{d^{2}y(t)}{dt^{2}}+\frac{c}{m}\frac{dy(t)}{dt}+\frac{2(\sigma_{m1}-\sigma_{m2})}{m\Delta^{3}}y(t)\approx \frac{F}{m}\cos\omega t+\frac{\sigma_{m1}-\sigma_{m2}}{m\Delta^{2}}.$$

The solution to the differential Equation (4) is sufficiently investigated in the *Theory of Vibrations with Application* book [26] and could be expressed as follows:

(5) $$y(t)=y_{h(t)}+y_{p(t)},$$

where $y_{h(t)}$ is the homogeneous solution and $y_{p(t)}$ stands for the particular solution.

After determining the particular solution for *y*(0) = 1 and *y*′(0) = 0, the final solution for (4) is given in (6).

(6) $$\begin{array}{l} y(t)=Ae^{(-\frac{c}{2m}-\sqrt{{\left(\frac{c}{2m}\right)}^{2}-\frac{2(\sigma_{m1}+\sigma_{m2})}{m\Delta^{3}}})t}+Be^{(-\frac{c}{2m}+\sqrt{{\left(\frac{c}{2m}\right)}^{2}-\frac{2(\sigma_{m1}+\sigma_{m2})}{m\Delta^{3}}})t}\\ +E\cos(\omega t-\eta )+\frac{\Delta (\sigma_{m1}-\sigma_{m2})}{2(\sigma_{m1}+\sigma_{m2})} \end{array}$$

where,

{(7)A=(c2m)2−2(σm1+σm2)mΔ3−c2m2(c2m)2−2(σm1+σm2)mΔ3,(8)B=(c2m)2−2(σm1+σm2)mΔ3+c2m2(c2m)2−2(σm1+σm2)mΔ3,(9)E=F(cω)2+[mω2−2(σm1+σm2)Δ3]2,(10)η=tan−1[−cωmω2−2(σm1+σm2)Δ3]

Equation (9) can be expressed in non-dimensional form as:

(11) $$\frac{E_{k}}{F}=\frac{1}{\sqrt{{\left[1-{\left(\frac{\omega }{\omega_{n}}\right)}^{2}\right]}^{2}+{\left[2\xi \frac{\omega }{\omega_{n}}\right]}^{2}}}$$

where,

(12) $$\left\{ \begin{array}{l} \omega_{n}=\sqrt{\frac{2(\sigma_{m1}+\sigma_{m2})}{m\Delta^{3}}}\\ \xi =\frac{c}{2m}\sqrt{\frac{m\Delta^{3}}{2(\sigma_{m1}+\sigma_{m2})}} \end{array}\right..$$

## 4. Dynamic Simulation of Electromagnetic Energy Harvester

**Figure 5:**
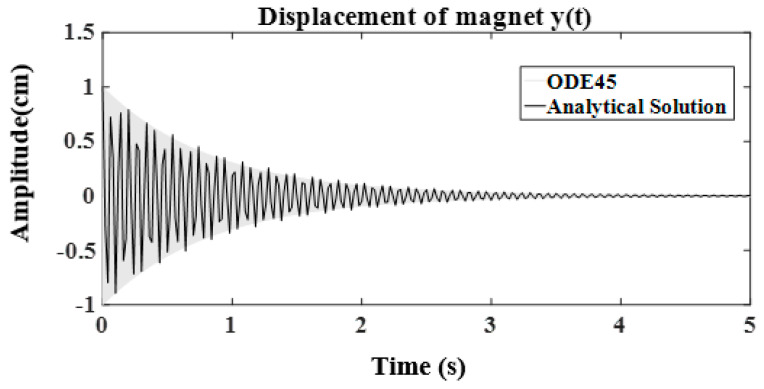
Displacement of central magnet using ODE45 and classical method for ODE.

**Figure 6:**
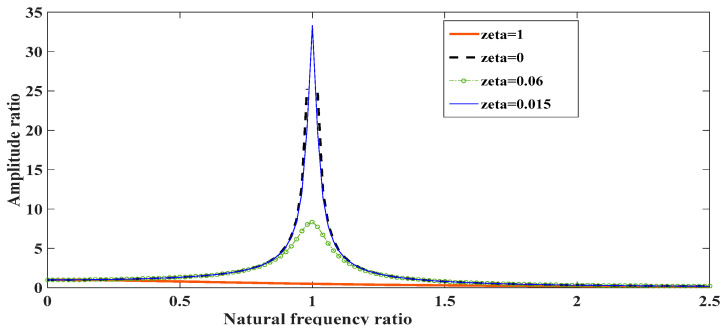
Amplitude ratio vs. frequency ratio response curve.

In this section, the dynamic system of the harvester is studied through numerical simulation with Matlab and ODE45 solver. Considering the construction parameters from Table 2 and some assumptions, the typical motion profile of the central magnet and the response frequency are given to illustrate the dynamic behavior of the harvester. (Figure 5) shows the motion profile of the central magnet, which is enabled by an initial excitation force (F) and decays due to the influence of electromechanical damping. The magnitude increases and decreases during the decaying oscillation of the central magnet. (Figure 6) shows the response frequency.

For frequency ratio $0.8\leq \frac{\omega }{\omega_{n}}\leq 1.2$, the harvester is near its resonance frequency and the maximum power can be harvested. For ζ greater than 0.06, the harvester exhibits anti-resonance behavior, and the generated power is minimal.

## 5. Experimental Verifications

**Figure 7:**
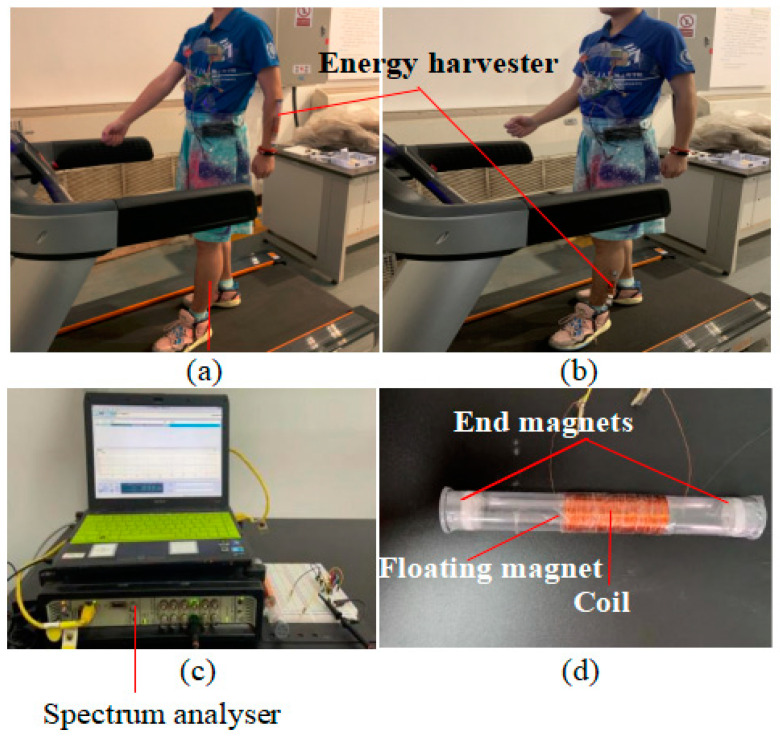
The measurement process of the voltage produced by the harvester, with the harvester attached to the arm (**a**) or lower extremity (**b**). The spectrum analyzer (**c**) and Harvester (**d**).

Experiments are conducted in two stages. In first stage, the capability of the harvester to sustain the power of the experimental prototype is evaluated (Figure 7). And in the second stage, the effectiveness of the self-built experimental IoT heartbeat rate-monitoring device is evaluated. The harvester is attached to the arm or lower extremity, as illustrated in Figure 7a,b. The treadmill speed is set to 3 mph (miles per hour), which corresponds to the average walking speed of a human being. The spectrum analyzer in Figure 7c is used for measuring the experimental voltage while walking on the treadmill.

▪ Voltage produced by the harvester

**Figure 8:**
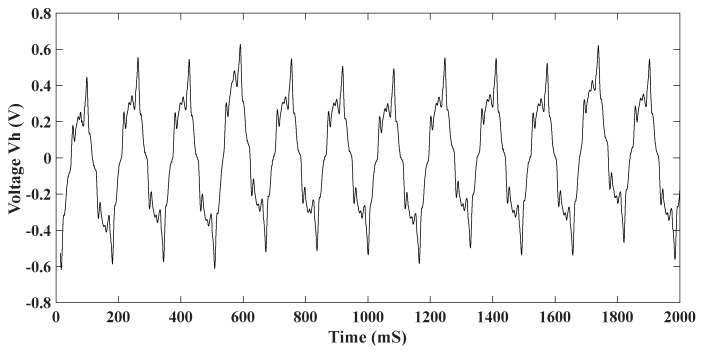
Voltage $V_{h}$ generated by the harvester.

The harvester is disconnected from the load and from the voltage multiplier. Both ends of the coils are directly connected to the spectrum analyzer. The voltage generated by the harvester during normal walking is shown in (Figure 8).

The generated power profile of the harvester follows the excitation force, which is a sinusoidal form (*F cos ωt*). It is either positive or negative throughout the whole period. As the central magnet moves, the magnetic field around the coil constantly changes between the north and south poles. Due to non-linear magnetic field interaction and electromechanical damping, the generated power is not smooth, and the magnitudes are not uniform.

▪ Voltage U across the capacitor

**Figure 9:**
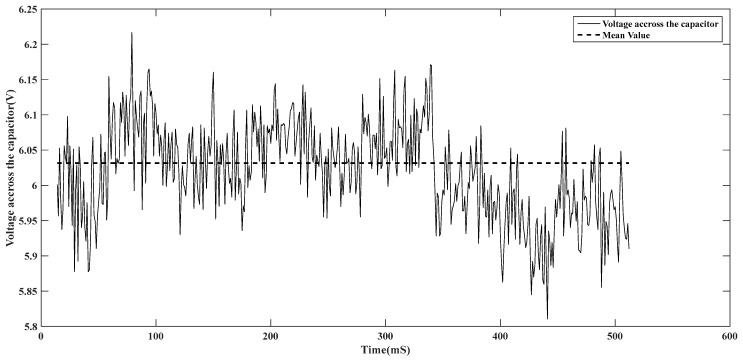
Voltage U across the capacitor.

The harvester is connected to the rectifier bridge, which multiplies the harvested voltage with a coefficient of k = 16. The voltage U in (Figure 9). is measured directly across the capacitor C.

The generated power from the harvester has passed though the bridge rectifier. Electronic switches enable us to reverse all the negative values and the capacitor for filtering the generated signal.

▪ Operating power of the experimental prototype of IoT-based heart rate-monitoring device.

**Figure 10:**
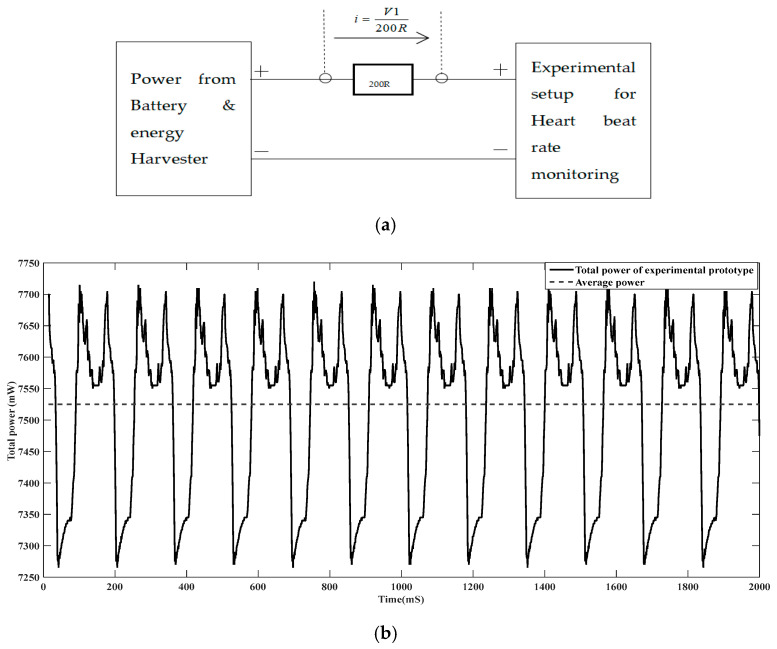
Measurement process (**a**), and experimental prototype power for IoT-based heartbeat rate-monitoring device (**b**).

For this measurement, the whole experimental setup is set on normal working conditions. A 200R resistor is connected in series to the power wire of the experimental prototype as illustrated in (Figure 10a). The drop voltage “$V_{1}$” is measured across the resistor 200R and the power “P” in (Figure 10b) is calculated based on the formula P=u×i, where

u is the voltage of experimental prototype; and
 i  is the current flowing to the experimental prototype ($i=\frac{v_{1}}{200R}$).

▪ Harvested power

**Figure 11:**
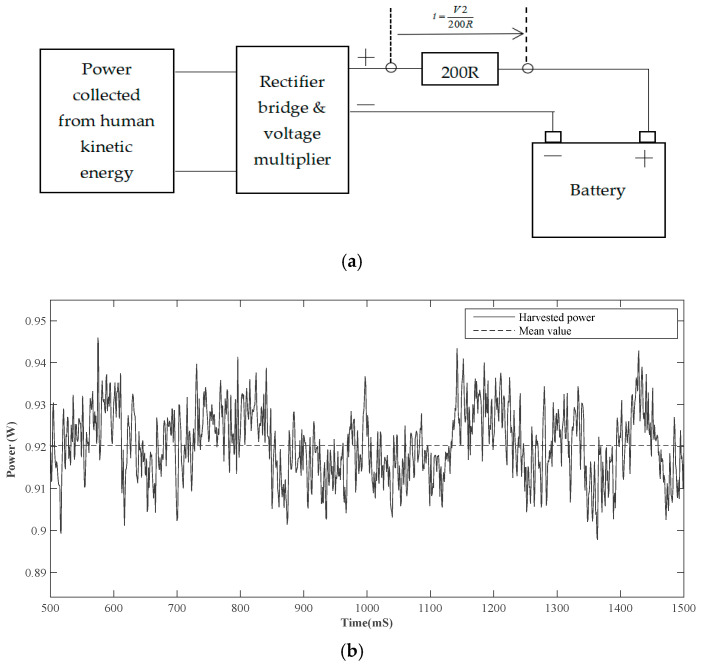
Measurement process (**a**); harvested power (**b**).

First the 3800 mAh battery is fully discharged and connected to the power rectifier, as illustrated in Figure 11a. A 200R resistor is placed in series with an electronic switch $D_{n}$ and the drop voltage $V_{2}$ is measured directly across the resistor. The harvested power (*P*) is calculated based on the formula P=u×i where

u is the voltage from rectifier bridge; and
i is the current flowing to the battery ($i=\frac{v_{2}}{200R}$).

When comparing the ratio of the power generated by the harvester over the power required by the whole experimental setup, the power generated by the harvester under normal walking conditions (Figure 11) is approximately 12.16% of the total power required by the whole experimental setup. Under running conditions, higher amplitude vibration energy can be harvested, which can better improve this result.

Furthermore, for a state-of-the-art manufacturing of such a system, deep investigations have to be conducted on the harvester to match the power harvested to the power required for operating the wearable device. Leveraging electronic design by using microelectronics devices can considerably lower the total power necessary for the IoT device.

### Experimental Setup for IoT-Based Heart Rate-Monitoring Device

**Figure 12:**
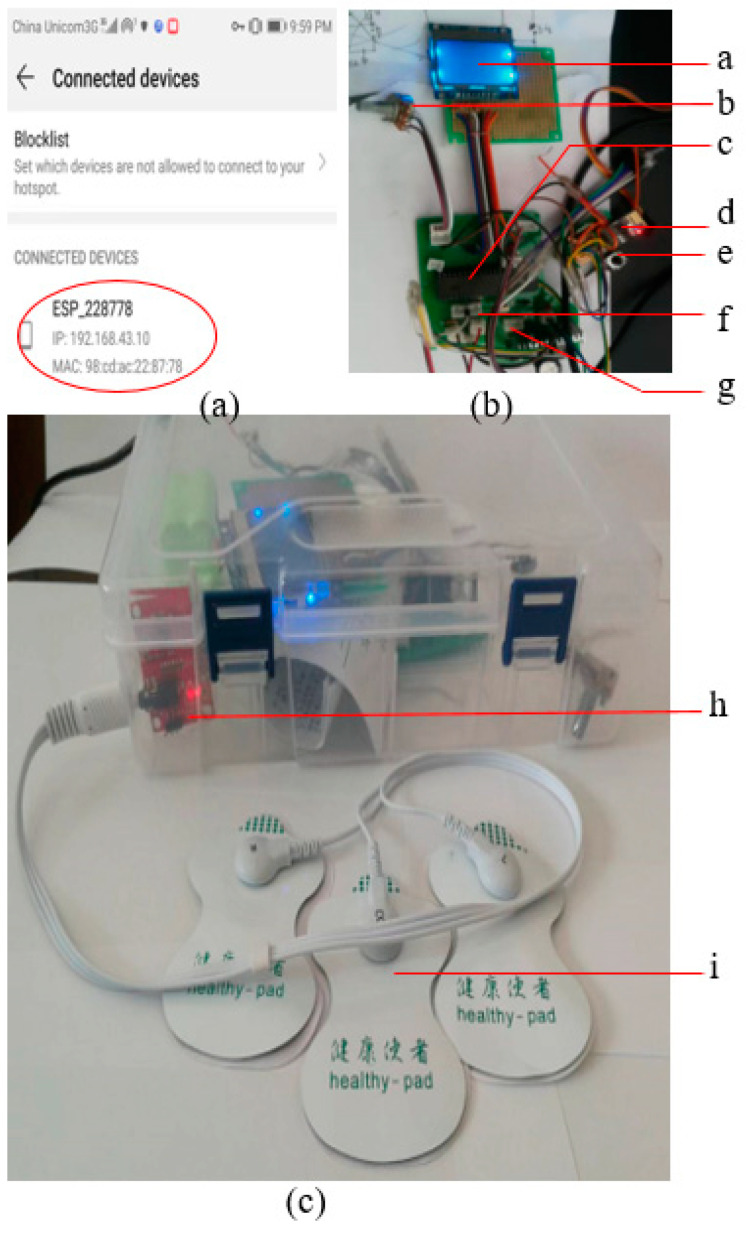
Experimental setup: using the mobile phone as Wi-Fi hotspot for connecting experimental setup (**a**); experimental setup connected to Wi-Fi (**b**). a: Screen; b: adjust contrast for screen; c: microcontroller; d: Wi-Fi serial transceiver; e: reset switch; f: pins for ECG sensor; g: power pins for harvester; (**c**) h: ECG sensor; i: ECG electrodes.

A personal space is created on the Matlab server “https://thingspeak.com/, accessed on 25 June 2024” for storing the heartbeat rate. The IP address of the server and channel ID are used in the code to access the host computer. The Pickit 3 device is used for transferring the code to the microcontroller. A personal mobile phone is used as Wi-Fi hotspot for connecting the experimental prototype to Wi-Fi. (Figure 12a) shows the experimental prototype connected to Wi-Fi and ready to share the health data. Different parts of the experimental setup are shown in (Figure 12b), and (Figure 12c) shows the overall setup with ECG electrodes.

▪ IoT Server

The IoT data server is a host computer, it is a highly reliable computer with a non-programming, data integration software. It is used for health data collection, processing, saving, notice, and sharing with remote scientists. This experiment makes use of an existing server, which is the Matlab server available at “https://thingspeak.com/, accessed on 25 June 2024” by creating a personal space for online data storage and retrieval.

▪ Microcontroller

The microcontroller is used to connect and coordinate all components (Wi-Fi device, display, and ECG electrodes). The microcontroller transmits AT commands to the Wi-Fi serial transceiver (ESP8266). The microcontroller used is PIC18F4550, which is a high-end, 8-bit microcontroller developed by Microchip Technology Inc, a publicly listed American corporation. It is one of the most available and low-cost microcontrollers (nearly 2.21 USD). Compared to other mid-range microcontrollers, it has serial communication pins, analog to digital conversion pins, high computational performance, high endurance, and enhanced flash program memory.

▪ Wi-Fi serial transceiver module (ESP8266)

The chip was developed by Espressif Systems Shanghai Co., Ltd. (Shanghai, China), a public multinational, fabless semiconductor company with their head office in Shanghai, China. In this study, it is used for interfacing the microcontroller and the IoT server. In fact, the Wi-Fi device receives health data from the microcontroller in the form of digital signal and transmits it to the IoT server, which enables remote scientists or cardiology hospitals to access the health data. Compared to the existing wireless technologies, ESP8266 is cost-effective (nearly 1 USD), and has low power consumption and a simple instruction set.

▪ Heartbeat rate sensor (AD8232)

AD8232 is a cost-effective ECG analog sensor (4.14 USD). Compared to other types of heartbeat rate sensor technologies, AD8232 has an integrated signal-conditioning block and ultra-low power consumption, which make it a suitable solution for wearable applications.

**Figure 13:**
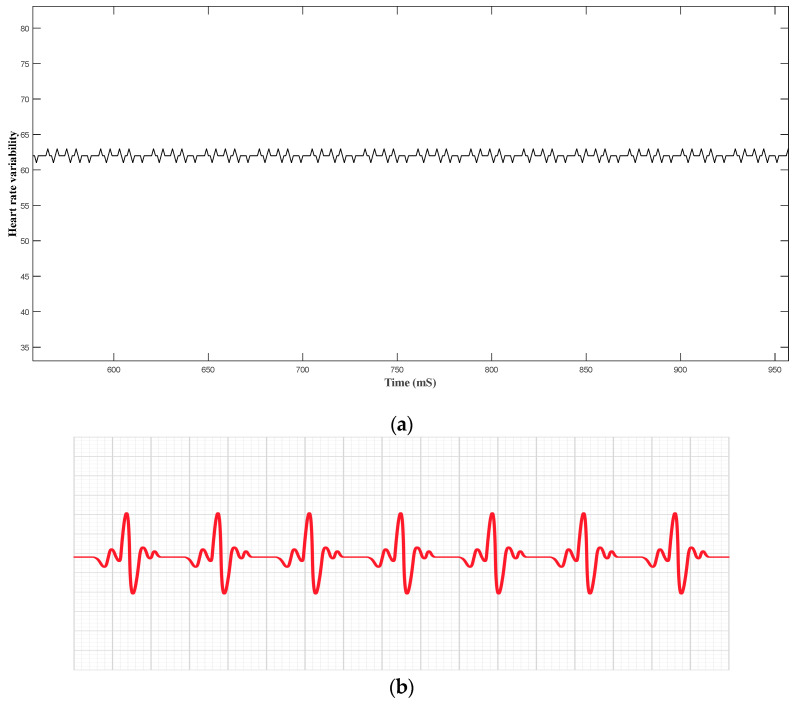
Experimental ECG wave form (**a**); conventional ECG wave form (**b**).

From Figure 13a, the wave progression does not differ throughout the whole period, which means the patient’s heart rhythm is regular.

## 6. Conclusions

In this work, the feasibility of using harvested human kinetic energy to sustain the power of biomedical devices is studied. The experimental prototype is based on a self-built IoT-based heartbeat rate-monitoring device for the remote monitoring of patients living with unstable health conditions. A non-linear electromagnetic harvester is implemented, and the non-linear restoring force of the magnet has an inverse quadratic dependence on the separation distance between the magnets. The same approach was used in [24]. This research considers the case of electromechanical damping and applies MacLaurin expansion to transform the inverse quadratic non-linearity of magnetic force into polynomial form, which enables us to provide the law of motion for general cases. A typical numerical application is computed using Matlab 2015 software and ODE45 based on the construction parameters of the harvester and some reasonable assumptions. Experiments are conducted on energy harvesters and an experimental prototype of an IoT-based heart rate-monitoring device to evaluate the capability of the harvester to sustain the power of the wearable device. We found that under average human walking conditions of 1.4 m per second, the harvester can sustain approximately 12.16% of the total power required by the wearable device, which is relatively low. This result can be better-improved under running conditions since the amplitude of the excitation force is higher. Furthermore, for the state-of-the-art manufacture of such a system, deep investigations have to be conducted on the harvester structure to match the power produced by the harvester to the power required by the wearable device. Improving the electronic design by using microelectronic devices can considerably reduce the power required by the wearable device.

## Figures and Tables

**Table 1:** Magnetic force vs. displacement.

Magnetic Forces	$F_{m1}$	$F_{m2}$	$F_{mc}$ = $F_{m1}$ + $F_{m2}$
Position y = 0	$\frac{\sigma_{m1}}{{∆}^{2}}$	$-\frac{\sigma_{m2}}{{∆}^{2}}$	$\frac{\sigma_{m1}-\sigma_{m2}}{{∆}^{2}}$
Position y=∆	$F_{max}$	$-\frac{\sigma_{m2}}{4{∆}^{2}}$	$F_{max}$
Position y=−∆	$\frac{\sigma_{m1}}{4{∆}^{2}}$	$-F_{max}$	$-F_{max}$

**Table 2:** Experimental Parameters.

Parameters	Value	Unit
Delta (∆)	0.075	m
Force (F)	1.5	N
Mass (m)	0.0094	Kg
$\mathrm{Sigma}\;(\sigma_{m1}$)	20	/
$\mathrm{Sigma}\;(\sigma_{m2}$)	20	/

**Figure A1:**
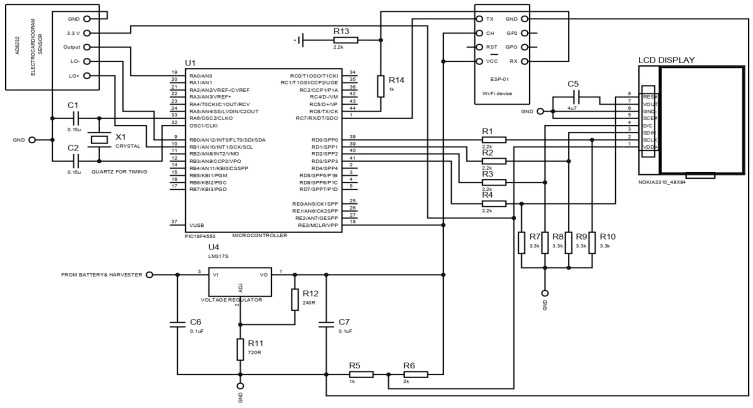
Schematic diagram illustrating the interconnection between electrocardiogram sensor, micro-controller, energy harvester and WI-FI device.

## Data Availability

Data are contained within the article.
